# Structures, Activities and Drug-Likeness of Anti-Infective Xanthone Derivatives Isolated from the Marine Environment: A Review

**DOI:** 10.3390/molecules24020243

**Published:** 2019-01-10

**Authors:** Daniela R. P. Loureiro, José X. Soares, Joana C. Costa, Álvaro F. Magalhães, Carlos M. G. Azevedo, Madalena M. M. Pinto, Carlos M. M. Afonso

**Affiliations:** 1 Laboratory of Organic and Pharmaceutical Chemistry, Department of Chemical Sciences, Faculty of Pharmacy, University of Porto, Rua de Jorge Viterbo Ferreira, 228, 4050-313 Porto, Portugal; dloureiro@ff.up.pt (D.R.P.L.); jcatarina@live.com.pt (J.C.C.); alfialma@hotmail.com (Á.F.M.); cgoncalves.azevedo@gmail.com (C.M.G.A.); madalena@ff.up.pt (M.M.M.P.); 2 Interdisciplinary Center of Marine and Environmental Investigation (CIIMAR/CIMAR), Edifício do Terminal de Cruzeiros do Porto de Leixões, Av. General Norton de Matos s/n, 4050-208 Matosinhos, Portugal; 3 Laboratory of Applied Chemistry, LAQV-REQUIMTE, Department of Chemical Sciences, Faculty of Pharmacy, University of Porto, Rua de Jorge Viterbo Ferreira, 228, 4050-313 Porto, Portugal; jxaviersoares@gmail.com

**Keywords:** marine, xanthones, antimicrobial, physicochemical properties, ADME

## Abstract

Marine organisms represent almost half of total biodiversity and are a very important source of new bioactive substances. Within the varied biological activities found in marine products, their antimicrobial activity is one of the most relevant. Infectious diseases are responsible for high levels of morbidity and mortality and many antimicrobials lose their effectiveness with time due to the development of resistance. These facts justify the high importance of finding new, effective and safe anti-infective agents. Among the variety of biological activities of marine xanthone derivatives, one that must be highlighted is their anti-infective properties. In this work, a literature review of marine xanthones with anti-infective activity, namely antibacterial, antifungal, antiparasitic and antiviral, is presented. Their structures, biological activity, sources and the methods used for bioactivity evaluation are described. The xanthone derivatives are grouped in three sets: xanthones, hydroxanthones and glycosylated derivatives. Moreover, molecular descriptors, biophysico-chemical properties, and pharmacokinetic parameters were calculated, and the chemical space occupied by marine xanthone derivatives is recognized. The chemical space was compared with marketed drugs and framed accordingly to the drug-likeness concept in order to profile the pharmacokinetic of anti-infective marine xanthone derivatives.

## 1. Introduction

Nowadays, infectious diseases are still one of the main causes of morbidity and mortality in the world [1]. *Per si*, this justifies the importance of seeking new antimicrobial agents. However, this problem is exacerbated with the growing emergence of drug resistant microbes. In fact, current anti-infective drugs are losing their efficacy at a rapid pace, and antimicrobial resistance has emerged as one of the major threats to public health in the 21st century [2].

Natural products (NPs) are evolutionarily optimized to be bioactive molecules, presenting a great chemical and pharmacological diversity, and they have always played a substantial role in drug discovery [3]. In fact, approximately sixty percent of the drugs approved in the last thirty years were from a natural origin or derived from natural products [4]. Amongst the natural sources, the marine environment contains the highest biodiversity of the planet [5,6]. Marine NPs have been discovered in a wide range of organisms, such as invertebrates or plants, and also microorganisms, such as fungi or bacteria [7]. Microorganisms can play an essential role in the discovery of new drugs, since they are responsible for the majority of bioactive secondary metabolites [7,8].

Marine organisms constitute a rich source of structurally diverse chemicals [9]. More than 28,000 molecules have been isolated from marine sources, among which more than 4,000 were bioactive [10]. Besides its richness, the marine environment provides a unique chemical diversity, as the use of specific biosynthetic pathways produces novel scaffolds quite different from those found in terrestrial sources [11]. Moreover, a myriad of attractive biological activities have been reported for marine NPs such as anticancer, antibacterial, antiviral, antifouling, and anti-inflammatory, etc. [12,13]. Among them, anti-infective (antibacterial, antifungal, antiparasitic, and antiviral) activities are one of the most frequent and with a great potential for developing new drugs [7]. Therefore, the exploitation of such a fertile environment opens and enlarges the potential for the discovery of novel and innovative anti-infective hits, leads and drugs.

**Figure 1:**
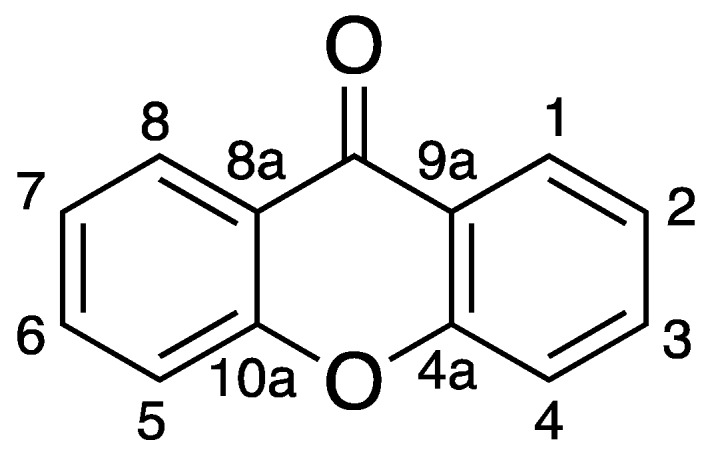
Xanthone Scaffold.

The most relevant chemical classes of secondary metabolites isolated from the marine biodiversity with antimicrobial activity are: terpenoids, peptides, steroids, alkaloids, polysaccharides, and polyketides [14,15]. Among the polyketides, xanthones are a class of oxygen-heterocycles containing a γ-pyrone moiety with two aromatic rings (Figure 1) [16]. They are considered “privileged structures” in Medicinal Chemistry, since depending on their chemical structure and the position of aromatic substituents, this family of compounds shows a variety of biological activities, such as antitumor, anti-inflammatory, antibacterial, and antifungal, among others [14,17,18,19].

Drug-like compounds are defined as those that have suitable pharmacodynamics and pharmacokinetics properties to become a drug [20]. Despite the clear definition, the materialization of drug-like chemical space is a difficult task. For this purpose, molecular descriptors are useful because they provide a numerical expression for chemical features. Descriptors such as molecular weight, number of hydrogens or number of rotatable bonds express features such as size, polarity, and flexibility [21]. The sum of several different molecular descriptors is used to establish a profiling of the key chemical features for a drug [22].

Besides molecular descriptors, biophysicochemical properties are also employed. The partition coefficient between octanol and water, known as Log P, is a remarkable example of the use of a physicochemical property to express a chemical feature. Log P is used to express lipophilicity which is a major determinant on the drug-likeness. In the pharmacodynamic behavior, an increase in lipophilicity might increase the potency, mostly due to contribution of entropically favored interactions between hydrophobic functionalities of the drug and the putative receptor [23]. However, excessively lipophilic compounds (log P > 3) show a great tendency for receptor promiscuity and eventually to toxicity [21]. Among the pharmacokinetic behavior, some lipophilicity is required to guarantee sufficient membrane permeability and renal clearance (log P > 0.8), whereas too lipophilic compounds (Log P > 4) tend to have a less favorable ADMET profile [24,25].

Over the years, sets of rules or filters were codified in order to help to define the drug-likeness chemical space. The most common criteria for drug-like chemical space are the Lipinski′s rule of five, which has gained widespread popularity [26]. Nevertheless, other approaches have been proposed by other authors, namely by Veber [27], Ghoose [28], Egan [29], and Muegge [30]. Despite the differences between these rules, the rationale behind them is the same: the definition of limits for molecular descriptors and/or physicochemical properties within compounds tend to have a suitable pharmacokinetic behavior. Due to its usefulness, these rules have paramount importance in drug discovery program as they to help medicinal chemists design molecules within the drug-likeness territory [31].

Gastrointestinal (GI) absorption [32], blood-brain barrier (BBB) permeation [32], cytochromes P450 [33] (CYP) inhibition, and the ability of being a substrate of the permeability glycoprotein (P-gp) [33] can be estimated before the substance is even synthesized and/or tested. These predictions are established by comparing the similarity between the tested compound and the large datasets of compounds known PK parameters. PK prediction allows the establishment of the profile of a class of compounds and acts as warning signals, highlighting at least for the need of a more detailed look at a given parameter [21].

Our group have been devoting special attention to xanthone derivatives, namely to their synthesis, PK behavior and biological activities [34,35,36]. Considering the anti-infective activity, several xanthone derivatives have been isolated from distinct natural sources [37,38,39,40,41,42]. In this work, we review the marine-derived xanthones with anti-infective activity described in the literature from 1989 to the present. For each xanthone derivative, the structure, the biological activity, the marine source and the methods used for biological activity evaluation are presented. Moreover, we evaluated the drug-likeness of all described xanthones derivatives considering molecular descriptors, biophysicochemical properties, and PK parameters. The obtained values were compared with those from marketed drugs and with the most common rules of drug-likeness guidelines. Finally, the general trends on the PK behavior prediction of this set of compounds was established.

## 2. Anti-Infective Xanthones Isolated from Marine Environment

The bibliographic survey was conducted using Scopus, Web of Science, PubMed, and Google Scholar. The keywords used were: marine xanthones, marine-derived xanthones, xanthones with antimicrobial activity, xanthones with anti-infective activity, xanthones with antibacterial activity and marine xanthones with antifungal activity, marine xanthones with antiviral activity and marine xanthones with antiparasitic activity. The survey covered the period between 1989 and 2018.

**Figure 2:**
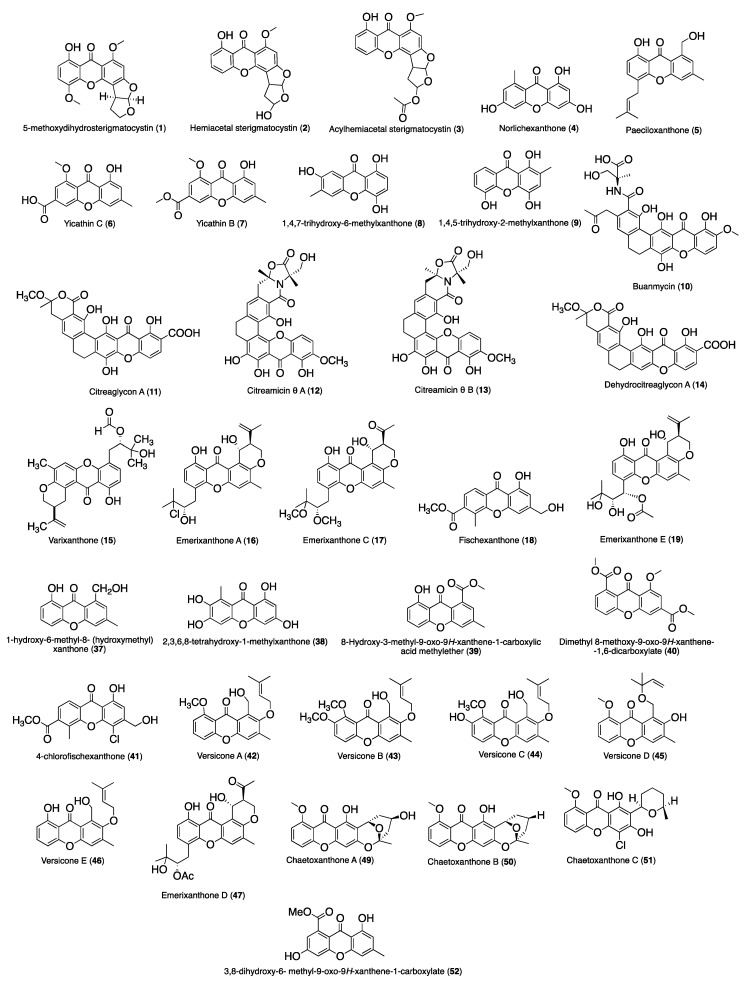
Structures of xanthone derivatives with anti-infective activity.

**Figure 3:**
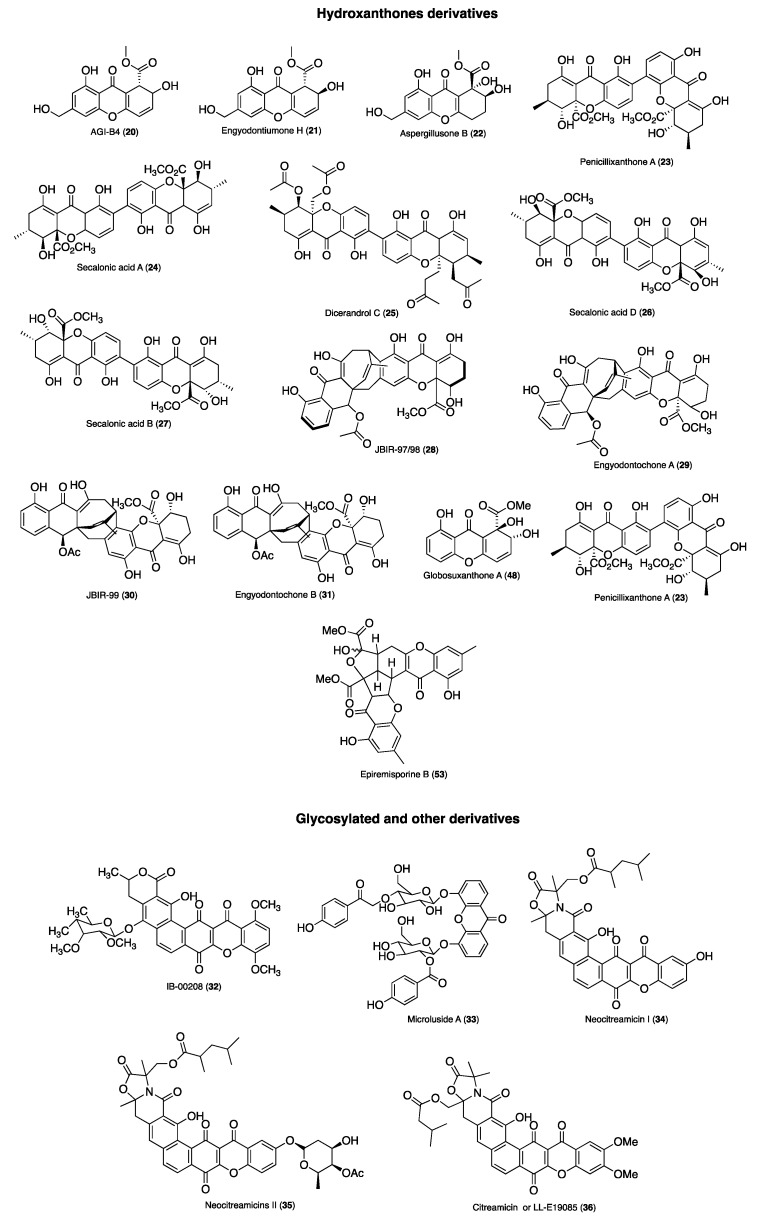
Structures of hydroxanthones, glycosylated and other derivatives with anti-infective activity.

Figure 2 and Figure 3 summarize the structures of the reported marine derived xanthones with antibacterial, antifungal, antiparasitic and antiviral activity. For each compound an identification number (ID) was assigned. Table 1 provides the compound name and ID, the anti-infective activity, the natural source, and the method used for the biological activity evaluation. Compounds were sorted into three categories: xanthones, hydroxanthones and glycosylated derivatives for each activity. Compounds bearing dibenzo-γ-pyrone scaffolds were classified as xanthone derivatives. Compounds bearing at least one saturated bond on the scaffold were classified as hydroxanthones derivatives. Compounds bearing at least one sugar moiety were classified as glycosylated derivatives. Neocitreamicins I and II (ID: 34 and 35, respectively) were grouped into the same category.

## 3. Comparison of Drug-Likeness of Marine Xanthone Derivatives with Marketed Drugs

Molecular descriptors of the identified marine anti-infective xanthone derivatives were calculated using SwissADME provided by the Swiss Institute of Bioinformatics [82]. For each compound, the following descriptors were calculated:

- molecular weight (MW),
- number of stereogenic centers,
- number of hydrogen bond acceptors (HBA) and donors (HBD), described as the electrostatic bond between a hydrogen and a lone pair of electrons,
- number of rotatable bonds (RB),
- number of rings,
- fraction of sp^3^ carbons (Fsp^3^) defined as the ratio of sp^3^ hybridized carbons over the total number of carbons [33]
- fraction of aromatic heavy atoms (FAr), defined as the number of aromatic heavy atoms divided by the total number of heavy atoms.

**Figure 4:**
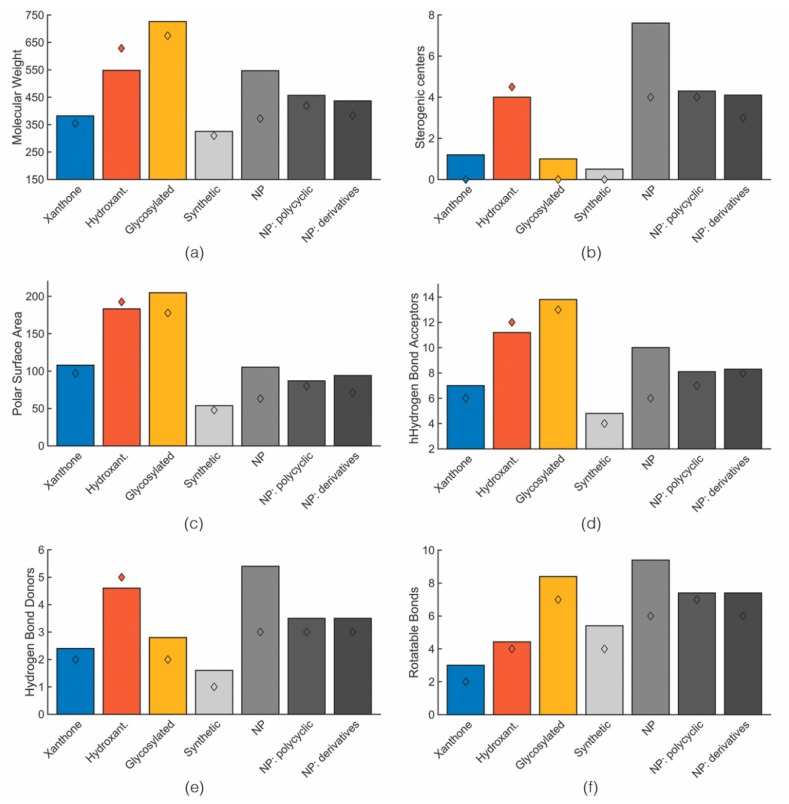
Mean (bar) and median (diamond) values of MW (**a**), stereogenic centers (**b**), PSA (**c**), HBA (**d**), HBD (**e**), rotatable bond (**f**) for marine xanthone derivatives (blue), hydroxanthone derivatives (orange), glycosylated derivatives (yellow), marketed drug types accordingly to its origin (greys).

Table S1 (Supplementary Materials) displays the obtained values for each molecular descriptor grouped accordingly to the categories defined in the previous section. Closely related to topological descriptors, biophysicochemical properties are quite important to target the sweet spot of both suitable pharmacodynamics and pharmacokinetics properties. Therefore, the following biophysico-chemical properties were calculated using SwissADME and ACDlabs [83]: polar surface area (PSA), log P, log D_7.4_ and log S. Table S2 (Supplementary Materials) shows the obtained values for each compound. For log P and log S, more than one algorithm was used in the calculation. For each category of the identified anti-infective marine xanthone derivatives, mean and median values of six molecular descriptors were calculated (Figure 4).

For the sake of comparison between the chemical space occupied by marine anti-infective xanthones and the marketed drugs, the obtained mean and median values were compared with marketed drugs, sorted accordingly to its origin: synthetic, natural products, and natural products derivatives (Figure 4) [26]. As xanthones are polycyclic compounds, values for drugs obtained from polycyclic NP, were also extracted and presented. A discussion involving the different molecular descriptors for marketed drugs and anti-infective xanthones is detailed in the following chapters. For this analysis the biological activity was not specified, because different methods were used to assess the same biological activity.

### 3.1. Size: Molecular Weight

According to the results presented in Figure 4a, the mean molecular weight for marine xanthone derivatives is 382.0 g·mol^−1^, for hydroxanthone derivatives 548.0 g·mol^−1^ and for gycosylated 726.1 g·mol^−1^. The higher molecular weight of gycosylated is expected due to the presence of, at least, one sugar moiety. The high molecular weight of hydroxanthone derivatives is attributed to the identification of several dimeric structures. The range of molecular weight presented by marine xanthone derivatives is different from the drug derived from natural products, mainly with the polycyclic natural products. Whereas xanthone derivatives are on average smaller, hydroxanthones are on average bigger than natural product drug.

Considering the Lipinski′s limit of molecular weight of 500, it is apparent that only xanthone derivatives adhere to it. However, it should be mentioned that antimicrobial drugs are typically larger and more complex than therapeutics from almost every other therapy area, except cancer [84]. Therefore, it is frequently to oral administered anti-infective drugs derived from natural products which do not obey to this Lipinski′s requirement.

### 3.2. Chirality: Number of Stereogenic Centers

Usually, natural products based drugs tend to have more stereogenic centers than drugs from synthetic origin (Figure 4b), because the use of stereospecific reagents and catalysts by the biological processes frequently lead to bioactive molecules with high numbers of chiral centers [85].

Among the identified marine xanthones derivatives, hydroxanthone derivatives have a higher number of stereogenic centers. In addition to the presence of stereogenic centers, several dimeric hydroxanthones show the existence atropisomerism. As depicted in Figure 4b, the number of stereogenic centers on hydroxanthone derivatives (mean value of 4.0) is on the same range of drugs derived from polycyclic natural products (mean value of 4.3). The higher number of stereogenic centers on hydroxanthones is attributed to higher molecular weight, but also to the presence of saturated bonds within the scaffold which can lead to the presence of stereogenic centers.

### 3.3. Polarity: PSA and HBD/HBA

Polarity was inferred by the number of hydrogen bond acceptor (HBA), the number of hydrogen bond donor (HBD), and PSA of the molecules [86]. Regarding HBD/HBA, the average numbers indicate that drugs from natural products have more acceptors and substantially more donors than drug from synthetic origin (Figure 4c–e). Among the different categories, glycosylated derivatives have the most hydrogen bond acceptors due to the presence of the sugar moiety. Comparing xanthone and hydroxanthone derivatives, the difference in terms of HBD and HBA can be justified by the difference in molecular weight (Figure 4d,e). As they are polyphenolic structures, the increase in size is accompanied by an increase in the HBA and HBD. As several marine xanthone derivatives have carboxylic acid groups or an easily hydrolysed ester, special caution should be taken when considering HBD and HBA. Carboxylic acids are simultaneous acceptors and donors. However, due to the in vivo deprotonation, they act solely as hydrogen bond acceptors.

**Figure 5:**
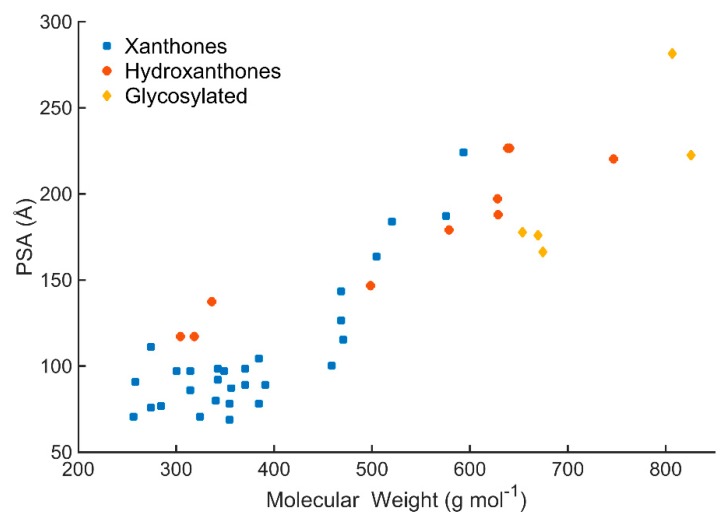
PSA values of the marine xanthone derivatives *vs* molecular weight (MW).

The PSA mean values were: 108.0 Å^2^ for xanthone derivatives, 183.2 Å^2^ for hydroxanthone derivatives, and 204.8 Å^2^ for glycosylated derivatives (Figure 4c). Similarly, to HBA/HBD, PSA values of the marine xanthone derivatives increases almost linearly with the increase in size (Figure 5). Applying the accepted limit of polar surface area which is 140 Å^2^, only xanthones with MW > 500 were able to fulfil this criterion.

Comparing with the PSA values of drugs originated from polycyclic natural, marine xanthone derivatives have higher PSA values (mean PSA of 108.0 vs. 86.9 for marine xanthones and polycyclic drugs, respectively) with lower molecular weight (mean MW of 382.0 vs. 456.7 for marine xanthones and polycyclic drugs, respectively) [26]. However, segmenting the chemical space of natural products and natural products-derived drugs according to the therapeutic area, anti-infective natural drugs are significantly more polar than their counterparts from other therapeutic areas, as reflected by their higher mean PSA (182.95 Å^2^) [84]. Therefore, the apparent excessive polarity of marine xanthone derivatives might be due to bias originating from the anti-infective activity.

### 3.4. Molecular Flexibility: Rotatable Bonds and Aromatic Character

The number of rotatable bonds (RB) is often used as a metric for molecular flexibility [27]. On the other hand, aromatic character, inferred by Fsp^3^ and Far, also expresses the molecular flexibility.

Among the marine xanthone derivatives, glycosylated xanthones have a high number of freely rotating bonds due to the presence of a sugar moiety. Xanthones and hydroxanthones have mean RB lower than the value found for drugs from polycylic natural products (Figure 4f). Marine hydroxanthones derivatives have an RB near the number found on synthetic drugs and have high number of rings, with a median value of six rings. In fact, half of the identified marine hydroxanthones have two additional rings. The data obtained suggest that at least some of these marine compounds are able to explore the thermodynamic advantages conferred by rigidity of the xanthone moiety, while retaining some flexibility in the attached rings.

Higher Fsp^3^ values are more a typical trait of natural products (mean Fsp^3^ of 0.55) than synthetic compounds (mean Fsp^3^ of 0.27) [21]. Regarding the marine xanthone derivatives, xanthones have a mean Fsp^3^ of 0.26, hydroxanthones have mean Fsp^3^ of 0.36, and the glycosylated ones have mean Fsp^3^ of 0.36. The obtained mean Fsp^3^ values are lower than the ones frequently found in natural products, namely in the case of xanthone derivatives. However, it should be highlighted that sp^3^ carbon atoms on natural products were likely to be part of the core scaffold [21]. As the xanthone moiety does not have any sp^3^ carbons, the influence of Fsp^3^ on biological activity can be biased. The other molecular descriptor for molecular aromatic character is the fraction of aromatic heavy atoms (Far). In this aspect, the obtained mean values for the marine xanthone derivatives were 0.54 for xanthones, 0.30 for hydroxanthones and 0.40 for glycosylated derivatives. In accordance with Fsp^3^ data, xanthone derivatives have a higher aromatic character. In fact, the majority of the heavy atoms present in xanthone derivatives are aromatic.

### 3.5. Lipophilicity: Log P

**Figure 6:**
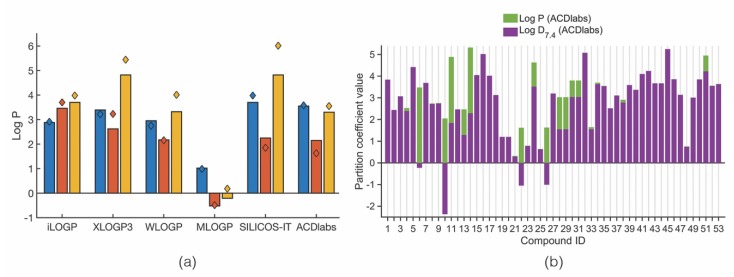
(**a**) Mean (bars) and median (diamonds) log P values of each category of marine xanthone derivatives calculated by different methods. (**b**) Difference between log P and log D_7.4_ calculated using ACDlabs.

Lipophilicity was assessed using log P and log D_7.4_. The obtained log P values of the marine xanthone derivatives vary a lot depending on the method used for the prediction. As shown in Figure 6a, the mean log P values differ from method to method, being the ones obtained with MLOGP the most discrepant. It can be assumed that for the majority of the identified compounds the log P value ranges between 2 to 4 units, in accordance with the mean log P found for drugs derived from polycyclic natural products [26]. However, considering just the anti-infective therapeutic area, orally bioavailable anti-infective drugs tend to have lower log P values than drugs from other therapeutic areas (mean log P of 1.56) [21].

For the case of xanthones bearing ionizable groups at physiological pH, log D_7.4_ is better metric to predict the in vivo lipophilicity [87]. Figure 6b shows the difference between log P and log D_7.4_ predicted by the same method (ACDlabs). Compounds showing only a purple column bar have the same log P and log D_7.4_. The remaining compounds have always a log D_7.4_ smaller than its corresponding log P value, due to the ionization at pH 7.4.

### 3.6. Solubility: Log S

Solubility is one of the most important properties in drug discovery. Low water solubility can lead to poor absorption and oral bioavailability, erratic assessment of the bioactivity, and confer additional challenges in later development stages [88]. Solubility is expressed as log S and values greater than −4 are acceptable for a drug [21].

**Figure 7:**
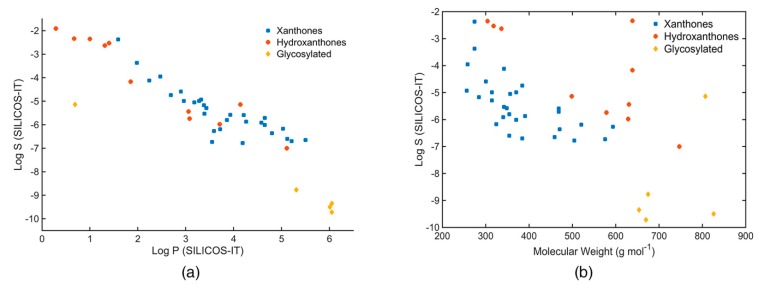
(**a**) Log S (SILICOS-IT) of the marine xanthone derivatives vs molecular weight. (**b**) Log S (SILICOS-IT) of the marine xanthone derivatives vs Log P (SILICOS-IT).

The relationship between molecular size and aqueous solubility of marine xanthone derivatives is fairly constant, except for microluside A which is a glycosylated xanthone (ID: **33**, Figure 7a). On the other hand, the aqueous solubility tends to decrease with increasing log P, independently of the type of scaffold (Figure 7b).

Considering the log S “rule of thumb” value of −4, the vast majority of the marine xanthone derivatives might face problems of solubility. In fact, a quite significant number of derivatives presented a log S lower than −6, which classifies them as poorly soluble molecules. Nevertheless, it should be mentioned that these solubility values do not take into account the ionization state. Therefore, at physiological pH, xanthone derivatives containing carboxylic acid groups should have a higher water solubility.

## 4. Compliance of Marine Xanthone Derivatives with the Rules of Drug-Likeness

The molecular descriptors and biophysicochemical properties of the identified xanthone derivatives were framed accordingly to the different preconized medicinal chemistry rules of drug-likeness (Table S3—Supplementary Materials). For each compound, violations of the different rules were evaluated as a percentage of compliance.

**Figure 8:**
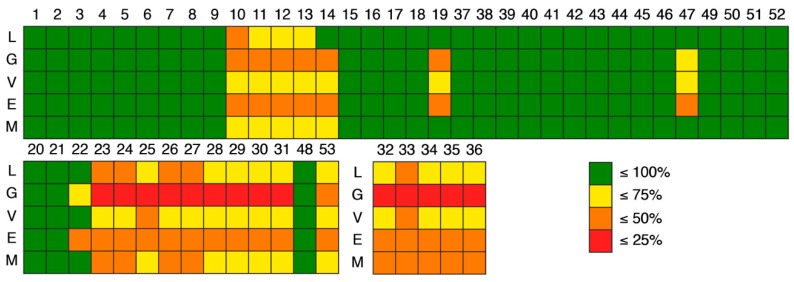
Colormap of the compliance with rules of drug-likeness: L—Lipinski, G—Ghose, V—Veber, E—Egan, M—Muegge. Xanthone derivatives (ID: 1–19, 37–47, 49–52) are represented on the upper quadrant, hydroxanthone derivatives (ID: 20–31, 48, 53) on the lower left and glycosylated derivatives (ID: 32–36) on the middle. Green means ≤100% of compliance, yellow means ≤75% of compliance, orange means ≤50% of compliance, and red means ≤25% of compliance.

For easier visualization a colormap (Figure 8) was applied: green for 100% of compliance, yellow for ≤75% of compliance, orange for ≤50% of compliance, and red for ≤25% of compliance, according to the rules of drug-likeness. As expected, the compliance was dependent of the threshold imposed by each rule. For example, for xanthone 19, the compliance for Lipinski and Muegge rule was ≤100%, for Veber the compliance was ≤75% and finally, for the Ghose and Egan rule was ≤50%. However, as general trend, the xanthone moiety appears to be more drug-likeness, as the compliances on this scaffold was higher than the ones obtained for hydroxanthones and glycosylated derivatives.

## 5. Trends on the PK Behavior of Marine Xanthone Derivatives

Gastrointestinal (GI) absorption and the ability to permeate the blood-brain barrier (BBB) were predicted using BOILED-Egg permeation method [32]. The compound ability to be a P-gp substrate or to inhibit one of five major isoforms of CYP450 (CYP1A2, CYP2C19, CYP2C9, CYP2D6, CYP3A4) were evaluated using SWISSADME [82]. The obtained results are presented in Table S4 (Supplementary Materials).

**Figure 9:**
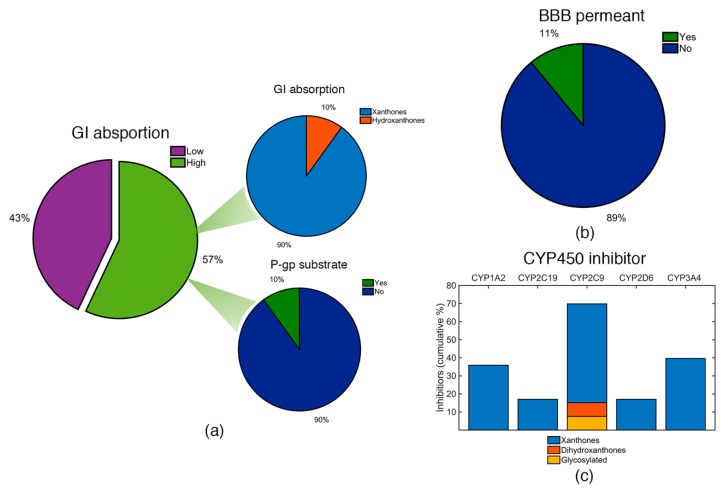
(**a**) GI absorption for the identified marine xanthone derivatives (left pie chart). Marine xanthone derivatives with high GI absorption were classified accordingly to its category (upper pie chart) and as P-gp substrate (lower pie chart). (**b**) BBB permeability of the identified xanthone derivatives. (**c**) Cumulative percentage of compounds identified as inhibitors of the five major isoforms of CYP450.

Considering GI absorption, the majority of the identified marine products have higher probability of being highly absorbed in the GI (Figure 9a). However, framing it in terms of the categories, the vast majority of the highly absorbed compounds belongs to the xanthone derivatives category (Figure 9a). This fact is attributed to their lower molecular size and lower polarity, when compared to hydroxanthones and glycosylated derivatives. Among the marine products with high GI absorption, the vast majority have low probability of being a substrate for P-gp (Figure 9a). All glycosylated derivatives were classified with low GI absorption and with high probability of effluxed by P-gp (Table S4—Supplementary Materials).

The majority of the identified compounds have a low probability of being able to across the BBB (Figure 9b). In fact, only six xanthone derivatives (ID: 4, 5, 37, 39, 42, and 45) have a good probability of being BBB permeants, precisely the derivatives with lowest PSA values (PSA < 78 Å^2^). It is noteworthy that any of these six derivatives have high probability of being a substrate of P-gp (Table S4—Supplementary Materials). Despite the relatively low BBB permeation, this is not a fundamental requirement for an anti-infective drug.

Regarding the CYP450 inhibition, marine products might be possible inhibitors of a CYP450 enzyme (Figure 9c). Among the different isoforms, CYP2C9 was the isoform with the highest probability to be inhibited, as almost 70% of the identified marine products were considered as possible inhibitors. In fact, it was the only isoform where hydroxanthone and glycosylated derivatives have been identified. The remaining isoforms were only inhibited by xanthone derivatives and several xanthones were identified as possible inhibitors of more than one CYP isoform (Table S4—Supplementary Materials).

## 6. Conclusions

Between 1989 and 2018, fifty-three marine xanthone derivatives with anti-infective activity (antibacterial, antifungal, antiparasitic and antiviral) were described in the literature. Most of them were isolated from microorganisms (mainly fungi) associated with macroorganisms (sponges or algae). As highlighted in Table 1, some xanthone derivatives have both antibacterial and antifungal activities, being the antibacterial activity predominant. Antibacterial xanthones present activity mainly against *Escherichia coli*, *Staphylococcus aureus*, and some are active against methicillin-resistant strains. Antifungal xanthones present activity mainly against *Candida albicans* and *Fusarium oxysporum*. However, the different methodologies used to evaluate the anti-infective activity hampers the comparison between different reports.

**Figure 10:**
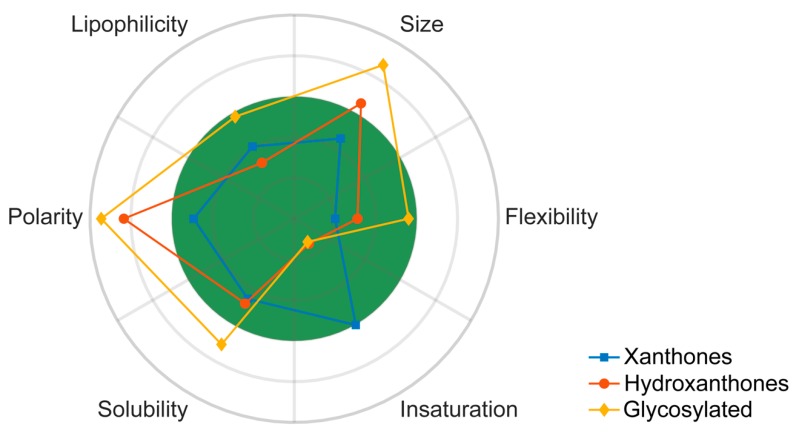
Polar plot of the marine xanthone chemical space. For each category, mean of RB (flexibility), mean of MW (size), mean of log P SILICOS-IT (lipophilicity), mean of PSA (Polarity), mean of log S SILICOS-IT (solubility), and mean Fsp^3^ (unsaturation) plotted in polar coordinates. Green colored zone: 0 < RB < 9; 150 < MW < 500; 0 < log P < 5; 20 < PSA < 130; -5 < log S < 0; 1 > Fsp^3^ > 0.25.

The drug-likeness of marine xanthone derivatives was evaluated using molecular descriptors, biophysicochemical properties and PK parameters, and it is summarized in Figure 10. Xanthone derivatives have a good compliance with the drug-likeness chemical space, justifying their probable high GI absorption and low substrate interaction with P-gp. However, its relatively high unsaturation and low flexibility might be a source of undesirable inhibition of CYP enzymes. Hydroxanthones tend to have a more flexible scaffold, but their size and mainly their polarity exceeds the desired values. A similar but exacerbated profile was verified for glycosylated derivatives. Consequently, it is possible to predict that hydroxanthones and glycosylated derivatives might have a poor pharmacokinetic behavior.

However, there are many examples of successful anti-infective drugs originated or inspired by natural products, which also do not obey the usual drug-likeness rules. Bearing this in mind, considering the urgent need for new antimicrobial agents, the marine environment might play a fundamental role, at least as a source of hits and leads for new drugs.

## Figures and Tables

**Table 1:** Anti-infective xanthone derivatives isolated from marine sources.

ID	Name	Activity	Method	Source	Ref.
antibacterial activity					
**1**	5-Methoxydihydrosterigmatocystin	*S*. *aureus* (ATCC 6538) (MIC, 12.5 μg/mL), *B*. *subtilis* (ATCC 6633) (MIC, 3.125 μg/mL), MRSA (MIC, >100 μg/mL), *P*. *aeruginosa* (ATCC 15692) (MIC, >100 μg/mL)	Antimicrobial Susceptibility Testing Standards outlined	*Aspergillus versicolor* MF359 isolated from a marine sponge sample of *Hymeniacidon perleve* collected from the Bohai Sea	[43]
**2**	Hemiacetal sterigmatocystin	*S*. *aureus* (ATCC 6538) (MIC, >100 μg/mL), *B*. *subtilis* (ATCC 6633) (MIC, >100 μg/mL), MRSA (MIC, >100 μg/mL), *P*. *aeruginosa* (ATCC 15692) (MIC, >100 μg/mL)			
**3**	Acylhemiacetal sterigmatocystin	*S*. *aureus* (ATCC 6538) (MIC, >100 μg/mL), *B*. *subtilis* (ATCC 6633) (MIC, >100 μg/mL), MRSA (MIC, >100 μg/mL), *P*. *aeruginosa* (ATCC 15692) (MIC, >100 μg/mL)			
**4**	Norlichexanthone (3,6,8-trihydroxy-1-methylxanthone or 1,3,6- trihydroxy-8-methyl-*9H*-xanthen-9-one)	*S*. *aureus* (ATCC 27154) (MIC, 12.5 µg/mL), *S*. *ventriculi* (ATCC 29068) (MIC, 25.0 µg/mL), *P*. *aeruginosa* (ATCC 25668) (MIC, 25.0 µg/mL)	Microdilution assay	*P*. *raistrikii* obtained from the sponge *Axinella* cf. corrugate or the mangrove endophytic fungus *Talaromyces* sp. ZH-154	[44,45,46]
		*B*. *agisterium* (inhibition zone 1 mm), *B*. *megaterium* (inhibition zone 1 mm)	Diffusion assay	*Wardomyces anomalu* isolated from the green alga *Enteromorpha* sp. (Ulvaceae) collected in the Baltic Sea	
**5**	Paeciloxanthone	*E*. *coli* (inhibitory zones of 12 mm)	Standard disk assay	*Paecilomyces* sp. was isolated from an estuarine mangrove from the Taiwan Strait	[47]
**6**	Yicathin C	*E*. *coli* (inhibition diameter 12.0 mm), *S*. *aureus* (inhibition diameter 7.5 mm)	Standard agar diffusion assay	*Aspergillus wentii* (red alga *Gymnogongrus flabelliformis*) collected from the coast of Pingtan Island, China	[48]
**7**	Yicathin B	*E*. *coli* (inhibition diameter 9 mm)			
**8**	1,4,7-Trihydroxy-6-methylxanthone	*E*. *coli* (MIC, 32 μg/mL), *P*. *aeruginosa* (MIC, 32 μg/mL), *S*. *aureus* (MIC, >64 μg/mL), *V*. *alginolyticus* (MIC, 32 μg/mL), *V*. *harveyi* (MIC, 32 μg/mL), *V*. *parahaemolyticus* (MIC, 32 μg/mL)	Microplate assay	*Talaromyces islandicus* EN-501, an endophytic fungus obtained from the fresh collected marine red alga *Laurencia okamurai*	[49]
**9**	1,4,5-Trihydroxy-2-methylxanthone	*E*. *coli* (MIC, 4 μg/mL), *P*. *aeruginosa* (MIC, 4 μg/mL), *S*. *aureus* (MIC, 8 μg/mL), *V*. *alginolyticus* (MIC, 4 μg/mL), *V*. *harveyi* (MIC, 8 μg/mL), *V*. *parahaemolyticus* (MIC, 4 μg/mL)			
**10**	Buanmycin	*S*. *aureus* (MIC, 10.5 μM), *B*. *subtilis* (MIC, 0.7 μM), *K*. *rhizophila* (MIC, 10.5 μM)*, S*. *enterica* (MIC, 0.7 μM), *P*. *hauseri* (MIC, 21.1 μM), *S*. *aureus* sortase A (IC_50_: 43.2 μM)	Microdilution assay	*Streptomyces* strain from a tidal mudflat in Buan, Republic of Korea.	[50]
**11**	Citreaglycon A	*S*. *haemolyticus* (MIC, 8.0 μg/mL), *S*. *aureus* UST950701-005 (MIC, 16 μg/mL), *B*. *subtillis* 769 (MIC, 8.0 μg/mL), *S*. *aureus* ATCC43300 (MIC, 8.0 μg/mL)	Microdilution assay	*Streptomyces caelestis* from Red Sea	[51]
**12**	Citreamicin θ A	*S*. *haemolyticus* (MIC, 0.5 μg/mL), *S*. *aureus* UST950701-005 (MIC, 1.0 μg/mL), *B*. *subtillis* 769 (MIC, 0.25 μg/mL), *S*. *aureus* ATCC43300 (MIC, 0.25 μg/mL)			
**13**	Citreamicin θ B	*S*. *haemolyticus* UST950701-004 (MIC, 0.5 μg/mL), *S*. *aureus* UST950701-005 (MIC, 1.0 μg/mL), *B*. *subtillis* 769 (MIC, 0.25 μg/mL), *S*. *aureus* ATCC43300 (MIC, 0.25 μg/mL)			
**14**	Dehydrocitreaglycon A	*S*. *haemolyticus* UST950701-004 (MIC, 8.0 μg/mL), *S*. *aureus* UST950701-005 (MIC, 16 μg/mL), *B*. *subtillis* 769 (MIC, 8.0 μg/mL)			
**15**	Varixanthone	*E*. *coli* (MIC, 12.5 µg/mL), *Proteus* sp. (MIC, 12.5 µg/mL), *B*. *subtilis* (MIC, 12.5 µg/mL), *S*. *aureus* (MIC, 12.5 µg/mL), *E*. *faecalis* (MIC, 50 µg/mL)	Method [52]	*Emericella variecolor* was isolated from a sponge (Porifera) collected in the Caribbean Sea	[53]
**16**	Emerixanthone A	*E*. *coli* (ATCC 29922), *K*. *pneumoniae* (ATCC 13883), *S*. *aureus* (ATCC 29213), *E*. *faecalis* (ATCC 29212), *A*. *bacterbaumannii* (ATCC 19606), *A*. *hydrophila* (ATCC 7966): Diameters of inhibition zones were all 4–6 mm	Filter paper disc agar diffusion method	*Emericella* sp. SCSIO 05240 from South China Sea	[54]
**17**	Emerixanthone C	*E*. *coli* (ATCC 29922), *K*. *pneumoniae* (ATCC 13883), *S*. *aureus* (ATCC 29213), *E*. *faecalis* (ATCC 29212), *A*. *bacterbaumannii* (ATCC 19606), *A*. *hydrophila* (ATCC 7966): Diameters of inhibition zones were all 4–6 mm			[54,55,56]
**18**	Fischexanthone	*E*. *coli* (MIC, >1265.82 µM), *S*. *aureus* (MIC, >1265.82 µM)	Broth tube dilution method	Mangrove endophytic fungus *Alternaria* sp. R6 collected from the mangrove in Leizhou peninsula, Guangdong Province, China	[57]
**19**	Emerixanthone E	*E*. *coli* (ATCC 29922), *K*. *pneumoniae* (ATCC 13883), *S*. *aureus* (ATCC 29213), *E*. *faecalis* (ATCC 29212), *A*. *baumannii* (ATCC 19606), and *A*. *hydrophila* (ATCC 7966): Diameters of the inhibition zones ranged between 9 and 11 mm	Diffusion method	Marine fungus *Emericella* sp. was isolated from the South China Sea	[58]
**20**	AGI-B4	*E*. *coli* (zone of inhibition 13.8 mm), *B*. *subtilis* (zone of inhibition 16.5 mm)	Standard disc diffusion assay	*Engyodontium album* DFFSCS021 from a marine sediment sample collected in the South China Sea	[56]
		*E*. *coli* (MIC, 64 μg/mL), *B*. *subtilis* (MIC, 64 μg/mL)	Microbroth dilution method		
**21**	Engyodontiumone H	*E*. *coli* (zone of inhibition 15.8 mm), *B*. *subtilis* (zone of inhibition 17.5 mm)	Standard disc diffusion assay		
		*E*. *coli* (MIC, 64 μg/mL), *B*. *subtilis* (MIC, 32 μg/mL)	Microbroth dilution method		
**22**	Aspergillusone B	*E*. *coli* (zone of inhibition 11.0 mm), *B*. *subtilis* (zone of inhibition 14.4 mm)	Standard disc diffusion assay		
		*E*. *coli* (MIC, 64 μg/mL*), B*. *subtilis* (MIC, 64 μg/mL)	Microbroth dilution method		
**23**	Penicillixanthone A	*B*. *subtilis* (MIC, 24.4 µg/mL), *E*. *coli* JVC1228 (MIC, 24.4 µg/mL), *M*. *luteus* UST950701-006 (MIC, 24.4 µg/mL), *P*. *nigrifaciens* UST010620-005 (MIC, 97.5 µg/mL)	Standard disc diffusion assay	*Penicillium* sp. SCSGAF 0023 isolated from South China Sea gorgonian coral *Dichotella gemmacea*	[59]
**24**	Secalonic acid A	*S*. *aureus* (ATCC 27154) (MIC 12.5 μg/mL), *E*. *coli* (ATCC 25922) (MIC 25 μg/mL), *S*. *ventriculi* (ATCC 29068) (MIC 12.5 μg/mL), *P*. *aeruginosa* (ATCC 25668) (MIC, 12.5 μg/mL)	-	*Talaromyces* sp. ZH-154 from the South-China Sea	[45]
**25**	Dicerandrol C	*S*. *aureus* (ATCC 6538) (MIC, 1/1.33 µg/mL), *S*. *saprophyticus* (ATCC 15305) (MIC, 2/2.66 µg/mL)	Microdilution broth method	Endophytic fungus *Phomopsis longicolla* isolated from the tropical red seaweed *Bostrychia radicans* from Brazil	[55]
**26**	Secalonic acid D	*B*. *subtilis* (MIC, 24.4 µg/mL), *E*. *coli* JVC1228 (MIC, 24.4 µg/mL), *M*. *luteus* UST950701-006 (MIC, 24.4 µg/mL), *P*. *nigrifaciens* UST010620-005 (MIC, 97.5 µg/mL)	Standard disc diffusion assay	*Penicillium* sp. SCSGAF 0023 isolated from South China Sea gorgonian coral *Dichotella gemmacea*	[59]
		*S*. *aureus* ATCC 29,213 (IC_50_ 7.19 μM), *M*. *tuberculosis* (IC_50_ 1.26 μM)	Standardized single disk method	Marine sponge-derived fungus *Aspergillus* sp. SCSIO XWS03F03	[60]
**27**	Secalonic acid B	*B*. *megaterium* (15 mm), *B*. *subtilis* (MIC, 97.5 µg/mL), *E*. *coli* JVC1228 (MIC, 97.5 µg/mL), *M*. *luteus* UST950701-006 (MIC, 97.5 µg/mL), *P*. *nigrifaciens* UST010620-005 (MIC, 390.5 µg/mL)	Standard disc diffusion assay	*Blennoria* sp. and *Penicillium* sp. SCSGAF 0023 isolated from South China Sea gorgonian coral *Dichotella gemmacea*	[59,61,62]
**28**	JBIR-97/98	*S*. *epidermidis* (IC_50_ 0.20 (±0.04) μM*),* MRSA (IC_50_ 0.19 (±0.02) μM*), P*. *acnes* (IC_50_ 11.0 (±1.3) μM)	Microbroth dilution method	LF069 was isolated from the marine sponge *Cacospinga scalaris* sampled at the Limski Fjord, Croatia and classified as *Engyodontium album*	[63]
**29**	Engyodontochone A	*S*. *epidermidis* (IC_50_ 0.19 (±0.04) μM*),* MRSA (IC_50_ 0.17 (±0.02) μM), *P*. *acnes* (IC_50_ 13.8 (±1.7) μM)			
**30**	JBIR-99	*S*. *epidermidis* (IC_50_ 0.21 (± 0.04) μM), MRSA (IC_50_ 0.25 (± 0.07)μM), *P*. *acnes* (IC_50_ 14.1 (±2.7) μM)			
**31**	Engyodontochone B	*S*. *epidermidis* (IC_50_ 0.22 (±0.03) μM), MRSA (IC_50_ 0.24 (±0.04) μM), *P*. *acnes* (IC_50_ 11.7 (±2.4) μM)			
**32**	IB-00208	*E*. *coli* (ATCC 10536) (MIC, >150 nM), *K*. *pneumonie* (ATCC 29665) (MIC, >150 nM), *P*. *aerigona* (ATCC 10145) (MIC, >150 nM), *B*. *subtilis* (ATCC 6051) (MIC, 1.4 nM), *S*. *aureus* (ATCC 6538P) (MIC, 1.4 nM), *M*. *luteus* (ATCC 9341) (MIC, 0.09 nM)	-	*Actinomadura* sp. collected from northern coast of Spain	[64]
**33**	Microluside A	*E*. *faecalis* JH212 (MIC, 10 μM), *S*. *aureus* NCTC 8325 (MIC, 13 μM)	Microdilution assay	*Micrococcus* sp. EG45 was cultivated from the Red Sea sponge *Spheciospongia vagabunda*	[62]
**34**	Neocitreamicin I	*B*. *subtilis* 1A1 (MIC, 0.06 μg/mL), MRSA NRS1 (MIC, 0.50 μg/mL), MRSA NRS2 (MIC, 0.12 μg/mL), MRSA NRS71 (MIC, 0.12 μg/mL), *E*. *faecalis* (VRE 51299) (MIC, 0.06 μg/mL), *E*. *faecalis* (VRE 51575) (MIC, 0.12 μg/mL), *E*. *coli* K-12 (MIC, >8.0 μg/mL)	Liquid growth medium	*Nocardia* strain (G0655) isolated from a sandy soil sample collected in Falmouth, Massachusetts (USA)	[65]
**35**	Neocitreamicins II	*B*. *subtilis* 1A1 (MIC, 0.12 μg/mL), MRSA NRS1 (MIC, 1.0 μg/mL), MRSA NRS2 (MIC, 0.50 μg/mL), MRSA NRS71 (MIC, 0.50 μg/mL), *E*. *faecalis* (VRE 51299) (MIC, 0.06 μg/mL), *E*. *faecalis* (VRE 51575) (MIC, 0.25 μg/mL), *E*. *coli* K-12 (MIC, >8.0 μg/mL)			
**36**	Citreamicin α or LL-E19085α	*E*. *coli* (MIC, >128 μg/mL), *K*. *pneumoniae* (MIC, >128 μg/mL), *Serratia* sp. (MIC, >128 μg/mL), *Citrobacter* sp. (MIC, >128 μg/mL), *P*. *aeruginosa* (MIC, >128 μg/mL), *S*. *aureus* (MIC, <0.06–0.12 μg/mL), *S*. *epidermidis* (MIC, <0.06 μg/mL), *Enterococcus* sp. (MIC, <0.06–0.12 μg/mL), *Streptococcus* sp. (MIC, <0.06 μg/mL), *S*. *pneumoniae* (MIC, <0.06 μg/mL), *B*. *fragilis* (MIC, 16 μg/mL), *B*. *thetaiotaomicron* (MIC, 4 μg/mL), *Clostridium perfringens* (MIC, <0.06 μg/mL), *C*. *difficile* (MIC, <0.06 μg/mL) [66]	Agar diffusion method	Marine *Micromonospora* sp. [67]	[66,67]
antifungal activity					
**4**	Norlichexanthone (3,6,8-trihydroxy-1-methylxanthone)	*C*. *albicans* (ATCC 10231) (MIC, 6.25 μg/mL), *A*. *niger* (ATCC 13496) (MIC, 25.0 μg/mL), *F*. *oxysporum* f. sp. *Cubense* (MIC, 50.0 μg/mL)	Agar diffusion assay	*P*. *raistrikii* (obtained from the sponge *Axinella* cf. *corrugate*) or *Talaromyces* sp. ZH-154 from the South-China Sea	[44,45,46]
		*E*. *repens* (inhibition zone 1 mm)		*Wardomyces anomalus* isolated from the green alga *Enteromorpha* sp. *(*Ulvaceae*)* collected in the Baltic Sea	[47]
**6**	Yicathin C	*C*. *lagenarium* (inhibition zone 11.0 mm)	Standard agar diffusion test	*Aspergillus wentii (*red alga *Gymnogongrus flabelliformis*) collected from the coast of Pingtan Island, China	[48]
**10**	Buanmycin	*C*. *albicans* (MIC, 21.1 μM), *A*. *fumigatus* (MIC, 84.3 μM)	Microdilution method	*Streptomyces* strain from a tidal mudflat in Buan, Republic of Korea	[50]
**18**	Fischexanthone	*F*. *graminearum* (MIC, 474.68 µM), *C*. *musae* (MIC, 474.68 µM)	Broth tube dilution method	Mangrove endophytic fungus *Alternaria* sp. R6 collected in Leizhou peninsula, Guangdong Province, China	[57]
**37**	1-Hydroxy-6-methyl-8-(hydroxymethyl) xanthone	*E*. *repens* (inhibition zone 2 mm), *U*. *violacea* (inhibition zone 2 mm)	Agar diffusion assay	Ulocladium botrytis (strain no. 193A4), isolated from the marine sponge *Callyspongia vaginalis*, collected from Dominica, Caribbean	[68]
**38**	2,3,6,8-Tetrahydroxy-1-methylxanthone	*M*. *violaceum* (inhibition zone 1 mm)	Agar diffusion assay	*Wardomyces anomalus* Brooks & Hansford *(*Microascaceae, As-comycetes)*,* isolated from the green alga *Enteromorpha* sp. collected around Fehmarn island in the Baltic Sea	[44]
**39**	8-Hydroxy-3-methyl-9-oxo-9*H*-xanthene-1-carboxylic acid methylether	*G*. *musae* (Rate of inhibition 53%), *P*. *cichoralearum* (Rate of inhibition 48%), *C*. *glocosporioides (*Rate of inhibition 28%), *B*. *graminearum* (Rate of inhibition 4.6%), *F*. *exysporum* (Rate of inhibition 9.5%)	Disk assay method	Co-culture broth of two mangrove fungi (strain No. K38 and E33) collected in South China Sea coast	[69,70,71]
**40**	Dimethyl 8-methoxy-9-oxo-9*H*-xanthene-1, 6-dicarboxylat	*F*. *oxysporum f*. sp. *Cubense* (MIC, 12.5 µg/mL)	-	*Penicillium* sp. (ZZF 32#) isolated from the South China Sea	[71,72]
**41**	4-Chlorofischexanthone	*F*. *graminearum* (MIC, 107 µM), *C*. *musae* (MIC, 214 µM)	Broth tube dilution method	Mangrove endophytic fungus *Alternaria* sp. R6 collected from the mangrove in Leizhou peninsula, Guangdong Province, China	[57]
**42**	Versicone A	*C*. *cutatum* (MIC, 32 μg/mL), *F*. *oxysporum* (MIC, 128 μg/mL), *M*. *oryzae* (MIC, >200 μg/mL)	Broth microdilution method	*Aspergillus versicolor* SCSIO 05879 collected from the Indian Ocean	[73]
**43**	Versicone B	*C*. *cutatum* (MIC, >200 μg/mL), *F*. *oxysporum* (MIC, >200 μg/mL), *M*. *oryzae* (MIC, >200 μg/mL)			
**44**	Versicone C	*C*. *cutatum* (MIC, >200 μg/mL), *F*. *oxysporum* (MIC, >200 μg/mL), *M*. *oryzae* (MIC, >200 μg/mL)			
**45**	Versicone D	*C*. *cutatum* (MIC, >200 μg/mL), *F*. *oxysporum* (MIC, >200 μg/mL), *M*. *oryzae* (MIC, >200 μg/mL)			
**46**	Variecoxanthone A	*C*. *cutatum* (MIC, >200 μg/mL), *F*. *oxysporum* (MIC, >200 μg/mL), *M*. *oryzae* (MIC, >200 μg/mL)			
**47**	Emerixanthones D	*Fusarium* sp., *Penicillium* sp., *A*. *niger*, *R*. *solani*, *F*. *sporium* f. sp. niveum, F. *sporium* f. sp. Cucumeris: Diameters of inhibition zones of which were both 3–4 mm	Filter paper discagar diffusion method	*Emericella* sp. SCSIO 05240 from South China Sea	[54]
**24**	Secalonic acid A	*C*. *albicans* (ATCC 10231) (MIC, 6.25 μg/mL), *A*. *niger* (ATCC 13496) (MIC, 6.25 μg/mL), *F*. *oxysporum* f. sp. *Cubense* (MIC, 12.5 μg/mL)	Microdilution assay	*Talaromyces* sp. ZH-154 from the South-China Sea	[45]
**27**	Secalonic acid B	*M*. *violaceum* (inhibition zone 13 mm)	Standard disc diffusion assay	*Blennoria* sp. and *Penicillium* sp. SCSGAF 0023 isolated from South China Sea gorgonian coral *Dichotella gemmacea*	[59,61]
**28**	JBIR-97/98	*C*. *albicans* (IC_50_ 4.6 (±0.5) μM*), T*. *rubrum* (IC_50_ 4.1 (±0.8) μM*)*	Microbroth dilution method	LF069 was isolated from the marine sponge *Cacospinga scalaris* sampled at the Limski Fjord, Croatia and classified as *Engyodontium album*	[63]
**29**	Engyodontochone A	*C*. *albicans* (IC_50_ 6.1 (±4.5) μM*), T*. *rubrum* (IC_50_ 6.0 (±1.7) μM*)*			
**30**	JBIR-99	*C*. *albicans* (IC_50_ 13.5 (±0.9) μM*), T*. *rubrum* (IC_50_ 5.3 (±1.0) μM*)*			
**31**	Engyodontochone B	*C*. *albicans* (IC_50_ 4.6 (±0.7) μM*)*, *T*. *rubrum* (IC_50_ 4.3 (±0.9) μM*)*			
**48**	Globosuxanthone A	*C*. *albicans* IFM 4954 (7 mm inhibition zone)	Paper disk method	*B*. *bassiana* TPU942, was isolated from a piece of an unidentified marine sponge collected at Iriomote Island	[74]
antiparasitic activity					
**49**	Chaetoxanthone A	*T*. *brucei rhodesiense* (strain STIB 900) (IC_50_ 4.7 μg/mL), *T*. *cruzi* (strain Tulahuen C4) (IC_50_ > 10 μg/mL), *L*. *dono*V*ani* (strain MHOM-ET-67/L82) (IC_50_ 5.3 μg/mL), *P*. *falciparum* (IC_50_ 3.5 μg/mL)	Modified [^3^H]hypoxanthine incorporation assay	*Chaetomium* sp. from the Greek alga originated from Kamari on the island Santorini.	[75,76,77]
**50**	Chaetoxanthone B	*T*. *brucei rhodesiense* (strain STIB 900) (IC_50_ 9.3 μg/mL), *T*. *cruzi* (strain Tulahuen C4) (IC_50_ 7.1 μg/mL), *L*. *dono*V*ani* (strain MHOM-ET-67/L82) (IC_50_ 3.4 μg/mL), *P*. *falciparum* (IC_50_ 0.5 μg/mL)			
**51**	Chaetoxanthone C	*T*. *brucei rhodesiense* (strain STIB 900) (IC_50_ 42.6 μg/mL), *T*. *cruzi* (strain Tulahuen C4) (IC_50_ 1.5 μg/mL), *L*. *dono*V*ani* (strain MHOM-ET-67/L82) (IC_50_ 3.1 μg/mL), *P*. *falciparum* (IC_50_ 4.0 μg/mL)			
antiviral activity					
**52**	3,8-Dihydroxy-6- methyl-9-oxo-9*H*-xanthene-1-carboxylate	A/FM-1/1/47 (H1N1) (IC_50_ 4.80 ± 1.28 μM), A/Puerto Rico/8/34 H274Y (H1N1) (IC_50_ 9.40 ± 1.96 μM), A/Aichi/2/68 (H3N2) (IC_50_ 5.12 ± 1.49 μM)	3-(4,5-Dimethylthiazol-2yl)-2,5-diphenyltetrazolium bromide (MTT) colorimetric assay	Mangrove-derived fungus *Diaporthe* sp. SCSIO 41011from *Rhizophora stylosa,* which was collected in Sanya city, Hainan Province, China	[78]
**23**	Penicillixanthone A	HIV-1 SF162 (10 μM, 90.86 ± 0.82%)	TZM-bl cells	Jellyfish-derived fungus *Aspergillus fumigatus*	[79]
**53**	Epiremisporine B	anti-EV71 (IC_50_ 19.8 μM), H3N2 (IC_50_ 24.1 μM)	CPE inhibition assay [80]	*Penicillium* sp. SCSIO Ind16F01 was isolated from a deep-sea sediment sample collected in the Indian Ocean	[81]

MIC: Minimum inhibitory concentration, IC_50_: Half maximal inhibitory concentration. *A. bacterbaumannii: Acineto bacterbaumannii; A. baumannii: Acinetobacter baumannii; A. fumigatus: Aspergillus fumigatus; A. hydrophila: Aeromonas hydrophila; A. niger: Aspergillus niger; B. agisterium: Bacillus agisterium; B. fragilis: Bacteroides fragilis; B. megaterium: Bacillus megaterium; B. subtilis: Bacillus subtilis; B. thetaiotaomicron: Bacteroides thetaiotaomicron; C. albicans: Candida albicans; C. difficile: Clostridium difficile; C. glocosporioides: Colletotrichum glocosporioides; C. lagenarium: Colletotrichum lagenarium; C. lunata: Curvularia lunata; C. musae: Calletotrichum musae; C. perfringens: Clostridium perfringens; E. coli: Escherichia Coli; E. faecalis: Enterococcus faecalis; E. repens: Eurotium repens; F. graminearum: Fusarium graminearum; F. oxysporum f.* sp. *cubense: Fusarium oxysporum f.* sp. *cubense ; F. oxysporum f.* sp. *cucumeris: Fusarium oxysporum f.* sp. *cucumeris; F. oxysporum f.* sp. *niveum: Fusarium oxysporum f.* sp. *niveum; F. oxysporum: Fusarium oxysporum; G. musae: Gloeosporium musae; K. pneumoniae: Klebsiella pneumoniae; K. rhizophila: Kocuria rhizophila; L. donovani: Leishmania donovani; M. luteus: Micrococcus luteus; M. violaceum: Microbotryum violaceum;* MRSA: Methicillin-resistant *Staphylococcus aureus; P. acnes: Propionibacterium acnes; P. aeruginosa: Pseudomonas aeruginosa; P. cichoralearum: Peronophthora cichoralearum; P. falciparum: Plasmodium falciparum; P. hauseri: Proteus hauseri; P. infestans: Phytophthora infestans; P. nigrifaciens: Pseudoalteromonas nigrifaciens; R. solani: Rhizoctonia solani; S. aureus: Staphyloccocus aureus; S. enterica: Salmonella enterica; S. epidermidis: Staphylococcus epidermidis; S. haemolyticus: Staphylococcus haemolyticus; S. pneumoniae: Streptococcus pneumoniae; S. ventriculi: Sarcina ventriculi; T. brucei rhodesiense: Trypanosoma brucei rhodesiense; T. brucei: Trypanosoma brucei; T. cruzi: Trypanosoma cruzi; U. violacea: Ustilago violacea; V. alginolyticus: Vibro alginolyticus; V. harveyi: Vibro harveyi; V. parahaemolyticus: Vibro parahaemolyticus*.

## Supplementary Materials

The supplementary materials are available online.
