# Supplementary material for: Structures, Activities and Drug-Likeness of Anti-Infective Xanthone Derivatives Isolated from the Marine Environment: A Review
Source: Molecules. 2019 Jan 10;24(2):243. doi: 10.3390/molecules24020243 (PMC6359551; doi:10.3390/molecules24020243)
Supplement: Supplementary file 1 [file molecules-24-00243-s001.pdf]

SUPPLEMENTARY MATERIALS for

Structures, activities and drug-likeness of anti-infective xanthone derivatives isolated from marine environment: a review

Daniela R. P. Loureiro [1,2,†], José X. Soares [3,†], Joana C. G. Costa [1], Álvaro F. Magalhães [1], Carlos Azevedo [1], Madalena M. M. Pinto [1,2], Carlos M. M. Afonso [1]

[1] Department of Chemical Sciences, Laboratory of Organic and Pharmaceutical Chemistry, Faculty of Pharmacy, University of Porto, Rua de Jorge Viterbo Ferreira, 228, 4050-313 Porto, Portugal
[2] Interdisciplinary Center of Marine and Environmental Investigation (CIIMAR/CIMAR), Edifício do Terminal de Cruzeiros do Porto de Leixões, Av. General Norton de Matos s/n, 4050-208 Matosinhos, Porto, Portugal
[3] LA*QV*-REQUIMTE, Department of Chemical Sciences, Laboratory of Applied Chemistry, Faculty of Pharmacy, University of Porto, Rua de Jorge Viterbo Ferreira, 228, 4050-313 Porto, Portugal
* Correspondence: cafonso@ff.up.com; Tel.: +351 22 042 8500
† These authors contributed equally to this work.

Table S1. Molecular descriptors of marine anti-infective xanthones.

ID	Molecular Formula	MW (g mol-1)	Stereogenic centers	Nº HBA	Nº HBD	Rotatable bonds	Nº rings	Fraction Csp³	FAr
				**Xanthone derivatives**					
**1**	$C_{19}H_{16}O_7$	356.33	2	7	1	2	3	0.32	0.54
**2**	$C_{18}H_{14}O_7$	342.30	0	7	2	1	5	0.28	0.56
**3**	$C_{20}H_{16}O_8$	384.34	0	8	1	3	5	0.30	0.50
**4**	$C_{14}H_{10}O_5$	274.27	0	5	2	1	3	0.27	0.30
**5**	$C_{20}H_{20}O_4$	324.37	0	4	2	3	3	0.25	0.58
**6**	$C_{16}H_{12}O_6$	300.26	0	6	2	2	3	0.12	0.64
**7**	$C_{17}H_{14}O_6$	314.29	0	6	1	3	3	0.18	0.61
**8**	$C_{14}H_{10}O_5$	258.23	0	5	3	0	3	0.07	0.74
**9**	$C_{14}H_{10}O_5$	258.23	0	5	3	0	3	0.07	0.74
**10**	$C_{30}H_{27}NO_{12}$	593.53	2	12	7	8	5	0.27	0.47
**11**	$C_{27}H_{20}O_{11}$	520.44	0	11	5	2	6	0.22	0.53
**12**	$C_{30}H_{25}NO_{11}$	575.52	4	11	5	2	7	0.30	0.48
**13**	$C_{30}H_{25}NO_{11}$	575.52	4	11	5	2	7	0.30	0.48
**14**	$C_{27}H_{20}O_{10}$	504.44	0	10	4	2	6	0.22	0.54
**15**	$C_{26}H_{28}O_8$	468.50	3	8	3	6	4	0.38	0.41
**16**	$C_{25}H_{27}ClO_6$	458.93	3	6	3	4	4	0.40	0.44
**17**	$C_{26}H_{30}O_8$	470.51	3	8	2	6	4	0.46	0.41
**18**	$C_{17}H_{14}O_6$	314.29	0	6	2	3	3	0.18	0.61
**19**	$C_{27}H_{30}O_9$	498.52	4	9	4	6	4	0.41	0.39
**37**	$C_{15}H_{12}O_4$	256.25	0	4	2	1	3	0.13	0.74
**38**	$C_{14}H_{10}O_6$	274.23	0	6	4	0	3	0.07	0.70
**39**	$C_{16}H_{12}O_5$	284.26	0	5	1	2	3	0.12	0.67

**40**	$C_{18}H_{14}O_7$	342.30	0	7	0	5	3	0.17	0.56
**41**	$C_{17}H_{13}ClO_6$	348.73	0	6	2	3	3	0.18	0.58
**42**	$C_{21}H_{22}O_5$	354.40	0	5	1	5	3	0.29	0.54
**43**	$C_{22}H_{24}O_6$	384.42	0	6	1	6	3	0.32	0.50
**44**	$C_{21}H_{22}O_6$	370.40	0	5	2	5	3	0.29	0.52
**45**	$C_{21}H_{22}O_5$	354.40	0	5	1	5	3	0.29	0.54
**46**	$C_{20}H_{20}O_5$	340.37	0	5	2	4	3	0.25	0.56
**47**	$C_{26}H_{28}O_8$	468.50	3	9	3	6	4	0.42	0.41
**49**	$C_{20}H_{18}O_7$	370.35	5	7	2	1	4	0.35	0.52
**50**	$C_{20}H_{18}O_6$	354.35	5	6	1	1	4	0.35	0.54
**51**	$C_{20}H_{19}ClO_6$	390.81	3	6	2	2	4	0.35	0.52
**52**	$C_{16}H_{12}O_6$	300.26	0	6	2	2	3	0.12	0.64
**Hydroxanthones**									
**20**	$C_{16}H_{14}O_7$	318.28	1	7	3	3	3	0.25	0.43
**21**	$C_{16}H_{14}O_7$	318.28	2	7	3	3	3	0.25	0.43
**22**	$C_{16}H_{16}O_8$	336.29	3	8	4	3	3	0.38	0.42
**23**	$C_{32}H_{30}O_{14}$	638.57	6	14	6	5	6	0.38	0.26
**24**	$C_{32}H_{32}O_{14}$	640.59	6	14	6	5	6	0.44	0.13
**25**	$C_{40}H_{42}O_{14}$	746.75	6	14	4	11	6	0.45	0.22
**26**	$C_{32}H_{30}O_{14}$	638.57	6	14	6	5	6	0.38	0.13
**27**	$C_{32}H_{30}O_{14}$	638.57	6	14	6	5	6	0.38	0.26
**28**	$C_{34}H_{30}O_{12}$	628.19	0	12	5	4	7	0.35	0.25
**29**	$C_{34}H_{30}O_{12}$	628.19	4	12	5	4	7	0.35	0.26
**30**	$C_{35}H_{32}O_{11}$	628.62	5	11	5	4	7	0.37	0.26
**31**	$C_{35}H_{32}O_{11}$	628.62	5	11	5	4	7	0.37	0.26
**48**	$C_{15}H_{12}O_7$	304.25	3	7	3	2	3	0.20	0.45
**53**	$C_{30}H_{26}O_{12}$	578.52	1	12	3	4	7	0.40	0.38
**Glycosylated and other derivatives**									
**32**	$C_{36}H_{34}O_{13}$	674.65	0	13	1	6	6	0.39	0.41
**33**	$C_{40}H_{38}O_{18}$	806.72	0	18	8	13	5	0.33	0.45
**34**	$C_{36}H_{31}NO_{11}$	653.63	0	11	2	6	7	0.33	0.42
**35**	$C_{44}H_{43}NO_{15}$	825.81	4	15	2	10	8	0.43	0.33
**36**	$C_{36}H_{31}NO_{12}$	669.63	0	12	1	7	7	0.33	0.41

Table S2. Biophysiochemical properties of marine anti-infective xanthones.

ID	Molar refractivity	PSA (Å2)	Log P (iLOGP)	Log P (XLOGP3)	Log P (WLOGP)	Log P (MLOGP)	Log P (SILICOS-IT)	Log P (ACDlabs)	Log $D_{7.4}$ (ACDLabs)	Log *S* (Ali)	Log *S* (ESOL)	Log *S* (SILICOS-IT)
						**Xanthone derivatives**						
**1**	92.93	87.36	3.14	3.05	2.89	0.85	3.18	3.84	3.84	-4.55	-4.24	-5.05
**2**	87.6	98.36	2.44	2.55	2.20	0.75	2.24	2.44	2.44	-4.26	-3.92	-4.12
**3**	97.33	104.43	2.70	3.13	2.77	0.76	2.69	3.07	3.07	-4.99	-4.37	-4.74
**4**	71.03	75.99	1.88	1.69	2.25	0.25	1.59	2.53	2.39	-2.90	-2.76	-2.37
**5**	96.83	70.67	3.51	4.56	3.81	2.23	5.03	4.42	4.42	-5.77	-4.96	-6.17
**6**	80.43	96.97	2.28	3.02	2.67	1.00	2.90	3.48	-0.23	-4.72	-3.94	-4.59
**7**	84.75	85.97	2.41	3.35	2.76	1.24	3.43	3.69	3.69	-4.83	-4.15	-5.29
**8**	71.03	90.90	1.91	2.81	2.37	0.57	2.47	2.74	2.72	-4.38	-3.76	-3.95
**9**	71.03	90.90	2.04	2.81	2.37	0.57	2.47	2.76	2.74	-4.38	-3.76	-3.95
**10**	153	224.06	1.76	2.57	2.24	-0.80	3.59	2.05	-2.37	-6.92	-4.96	-6.27
**11**	133.16	183.96	2.59	4.73	3.31	0.59	3.72	4.89	1.84	-8.32	-6.30	-6.19
**12**	152.13	187.20	2.38	4.16	2.18	0.28	3.55	1.29	2.47	-7.80	-6.25	-6.73
**13**	152.13	187.20	3.37	4.16	2.18	0.28	3.55	2.47	1.29	-7.80	-6.25	-6.73
**14**	131.14	163.73	3.04	5.09	3.60	1.49	4.19	5.32	2.29	-8.27	-6.44	-6.78
**15**	126.95	126.43	3.49	4.10	3.11	1.29	4.65	4.05	4.05	-6.46	-5.24	-5.71
**16**	126.43	100.13	3.97	4.79	4.17	2.14	5.50	5.02	5.02	-6.62	-5.76	-6.65
**17**	128.12	115.43	3.76	3.31	3.25	0.55	4.80	4.02	4.02	-5.41	-4.75	-6.36
**18**	84.39	96.97	2.82	2.49	2.09	0.97	3.28	3.13	3.13	-4.17	-3.61	-4.99
**19**	133.69	146.66	3.46	2.98	2.67	0.71	4.14	3.20	3.2	-5.72	-4.70	-5.14
**37**	73.11	70.67	2.34	2.64	2.30	1.12	3.32	3.11	3.11	-3.77	-3.57	-4.93
**38**	73.05	111.13	1.88	2.46	2.08	0.02	1.98	2.91	2.78	-4.44	-3.61	-3.37

**39**	78.26	76.74	2.83	3.38	2.75	1.54	3.38	3.59	3.59	-4.67	-4.09	-5.17
**40**	89.04	92.04	2.89	2.64	2.53	1.42	3.39	3.37	3.37	-4.22	-3.71	-5.53
**41**	89.4	96.97	2.88	3.12	2.74	1.21	3.93	4.10	4.1	-4.83	-4.20	-5.58
**42**	102.82	68.90	3.36	3.88	3.95	1.89	5.12	4.24	4.24	-5.02	-4.55	-6.60
**43**	102.82	78.13	3.43	3.85	3.96	1.57	5.21	3.67	3.67	-5.19	-4.62	-6.70
**44**	109.31	89.13	3.25	3.53	3.65	1.35	4.65	3.67	3.67	-5.09	-4.41	-6.01
**45**	103.12	68.90	3.60	3.83	4.30	1.89	5.12	5.24	5.25	-4.97	-4.52	-6.60
**46**	98.35	79.90	3.32	4.11	3.65	1.67	4.58	3.86	3.86	-5.49	-4.69	-5.91
**47**	128.4	143.50	2.92	2.81	2.51	0.51	4.21	3.14	3.14	-5.48	-4.51	-5.59
**49**	97.25	98.36	3.14	2.78	2.66	0.81	2.96	3.01	3.01	-4.50	-4.21	-4.99
**50**	96.09	78.13	3.43	3.75	3.69	1.61	3.86	3.85	3.85	-5.08	-4.73	-5.80
**51**	103.51	89.13	3.37	4.37	4.33	1.69	4.26	4.95	4.22	-5.96	-5.27	-5.87
**52**	80.28	96.97	2.43	3.02	2.45	0.73	2.90	3.56	3.55	-4.72	-3.94	-4.59
**Hydroxanthone derivatives**												
**20**	80.45	117.20	2.38	0.96	0.37	-0.49	1.40	1.20	1.2	-3.01	-2.54	-2.53
**21**	103.47	117.20	2.34	0.96	0.37	-0.49	1.40	1.20	1.2	-3.01	-2.54	-2.53
**22**	81.17	137.43	1.26	0.26	-0.60	-1.21	1.31	0.32	0.29	-2.71	-2.20	-2.63
**23**	155.3	226.58	3.79	3.80	2.16	-1.36	1.85	1.62	-1.05	-8.25	-6.06	-4.17
**24**	155.17	226.58	3.67	2.30	1.50	-1.82	0.29	0.79	0.78	-6.70	-5.03	-1.91
**25**	191.84	220.26	4.75	3.54	5.41	-0.07	5.11	4.63	3.52	-7.85	-6.14	-7.00
**26**	155.1	226.58	3.62	2.36	2.33	-1.90	0.67	0.64	0.63	-6.76	-5.05	-2.34
**27**	155.3	226.58	4.58	3.80	2.16	-1.36	1.85	1.63	-1.01	-8.25	-6.06	-4.17
**28**	158.55	197.12	4.28	3.57	3.52	0.16	3.06	3.03	1.55	-7.40	-5.93	-5.44
**29**	161.54	197.12	4.26	3.57	3.52	0.16	3.06	3.03	1.55	-7.40	-5.93	-5.44
**30**	161.54	187.89	3.15	3.76	3.93	0.83	3.71	3.80	3.04	-7.40	-6.04	-5.98
**31**	161.54	187.89	3.73	3.76	3.93	0.83	3.71	3.80	3.04	-7.40	-6.04	-5.98
**48**	75.36	117.20	2.70	1.08	0.03	-0.74	1.00	0.76	0.74	-3.13	-2.61	-2.35

**53**	142.3	179.03	3.90	2.91	1.77	0.13	3.08	3.64	3.63	-6.33	-5.28	-5.74
**Glycosylated and other derivatives**												
**32**	173.33	166.26	4.09	5.44	4.34	-0.23	5.31	5.08	5.08	-8.69	-7.36	-8.77
**33**	196.41	281.57	2.61	1.56	0.49	-2.39	0.69	1.65	1.55	-7.08	-5.30	-5.14
**34**	175.42	177.72	3.50	5.84	4.01	0.93	6.05	3.70	3.63	-9.34	-7.48	-9.35
**35**	214.95	222.48	4.31	6.09	4.11	0.48	6.01	3.55	3.54	-10.54	-8.38	-9.50
**36**	176.76	175.95	3.98	5.21	3.68	0.18	6.05	2.52	2.52	-8.65	-7.11	-9.72

Table S3. Violations of drug-likeness rules by the marine anti-infective xanthones. For each compound, the type of violations of each rule was described.

ID	Lipinski	Ghose	Veber	Egan	Muegge
			**Xanthone derivativies**		
**1**	0	0	0	0	0
**2**	0	0	0	0	0
**3**	0	0	0	0	0
**4**	0	0	0	0	0
**5**	0	0	0	0	0
**6**	0	0	0	0	0
**7**	0	0	0	0	0
**8**	0	0	0	0	0
**9**	0	0	0	0	0
**10**	MW>500, HBA>10, HBA>5	MW>480, MR>130	PSA>140	PSA>131.6	PSA>150, HBA>10, HBD>5
**11**	MW>500, HBA>10	MW>480, MR>130	PSA>140	PSA>131.6	PSA>150, HBA>10
**12**	MW>500, HBA>10	MW>480, MR>130	PSA>140	PSA>131.6	PSA>150, HBA>10
**13**	MW>500, HBA>10	MW>480, MR>130	PSA>140	PSA>131.6	PSA>150, HBA>10
**14**	0	MW>480, MR>130	PSA>140	PSA>131.6	PSA>150, HBA>10
**15**	0	0	0	0	0
**16**	0	0	0	0	0
**17**	0	0	0	0	0
**18**	0	0	0	0	0
**19**	0	MW>480, MR>130	PSA>140	PSA>131.6	0
**37**	0	0	0	0	0
**38**	0	0	0	0	0
**39**	0	0	0	0	0
**40**	0	0	0	0	0
**41**	0	0	0	0	0
**42**	0	0	0	0	0
**43**	0	0	0	0	0
**44**	0	0	0	0	0
**45**	0	0	0	0	0
**46**	0	0	0	0	0
**47**	0	MR>130	PSA>140	PSA>131.6	0
**49**	0	0	0	0	0
**50**	0	0	0	0	0
**51**	0	0	0	0	0

**52**	0	0	0	0	0
**Hydroxanthones derivatives**					
**20**	0	0	0	0	0
**21**	0	0	0	0	0
**22**	0	TNA>70	0	PSA>131.6	0
**23**	MW>500, HBA>10, HBA>5	MW>480, MR>130, TNA>70	PSA>140	PSA>131.6	MW>600, PSA>150, HBA>10, HBD>5
**24**	MW>500, HBA>10, HBA>5	MW>480, MR>130, TNA>70	PSA>140	PSA>131.6	MW>600, PSA>150, HBA>10, HBD>5
**25**	MW>500, HBA>10	MW>480, MR>130, TNA>70	PSA>140, RB>10	PSA>131.6	MW>600, PSA>150, HBA>10
**26**	MW>500, HBA>10, HBA>5	MW>480, MR>130, TNA>70	PSA>140	PSA>131.6	MW>600, PSA>150, HBA>10, HBD>5
**27**	MW>500, HBA>10, HBA>5	MW>480, MR>130, TNA>70	PSA>140	PSA>131.6	MW>600, PSA>150, HBA>10, HBD>5
**28**	MW>500, HBA>10	MW>480, MR>130, TNA>70	PSA>140	PSA>131.6	MW>600, PSA>150, HBA>10
**29**	MW>500, HBA>11	MW>480, MR>130, TNA>71	PSA>140	PSA>131.6	MW>600, PSA>150, HBA>10
**30**	MW>500, HBA>12	MW>480, MR>130, TNA>72	PSA>140	PSA>131.6	MW>600, PSA>150, HBA>10
**31**	MW>500, HBA>13	MW>480, MR>130, TNA>73	PSA>140	PSA>131.6	MW>600, PSA>150, HBA>10
**48**	0	0	0	0	0
**53**	MW>500, HBA>10	MW>480, MR>130	PSA>140	PSA>131.6	PSA>150, HBA>10
**Glycosylated and other derivatives**					
**32**	MW>500, HBA>10	MW>480, MR>130, TNA>70	PSA>140	PSA>131.6	MW>600, PSA>150, HBA>10, LOG P>5

**33**	MW>500, HBA>10, HBA>5	MW>480, MR>130, TNA>70	PSA>140, RB>10	PSA>131.6	MW>600, PSA>150, HBA>10, HBD>5
**34**	MW>500, HBA>10	MW>480, MR>130, TNA>70	PSA>140	PSA>131.6	MW>600, PSA>150, HBA>10, LOG P>5
**35**	MW>500, HBA>10	MW>480, MR>130, TNA>70	PSA>140	PSA>131.6	MW>600, PSA>150, HBA>10, HBD>5, LOG P>5
**36**	MW>500, HBA>10	MW>480, MR>130, TNA>70	PSA>140	PSA>131.6	MW>600, PSA>150, HBA>10, LOG P>5

MW – molecular weight, MR - molar refractivity, TNA - total number of atoms, PSA – polar surface area, HBA – hydrogen bond acceptor, HBD – hydrogen bond donor, RB - rotatable bonds, LOG P – considered log P value calculated by XLOGP3.

Table S4. Absorption and Metabolism parameters of marine antimicrobial xanthones.

ID	GI absorption	BBB permeant	P-gp substrate	CYP1A2 inhibitor	CYP2C19 inhibitor	CYP2C9 inhibitor	CYP2D6 inhibitor	CYP3A4 inhibitor
**Xanthone derivativies**								
**1**	High	No	No	Yes	Yes	Yes	Yes	Yes
**2**	High	No	No	Yes	No	No	Yes	No
**3**	High	No	No	No	No	Yes	Yes	No
**4**	High	Yes	No	No	No	No	No	No
**5**	High	Yes	No	Yes	Yes	Yes	No	Yes
**6**	High	No	No	Yes	No	Yes	No	No
**7**	High	No	No	Yes	No	Yes	No	No
**8**	High	No	No	Yes	No	No	Yes	Yes
**9**	High	No	No	Yes	No	No	Yes	Yes
**10**	Low	No	No	No	No	Yes	No	No
**11**	Low	No	No	No	No	Yes	No	No
**12**	Low	No	No	No	No	Yes	No	No
**13**	Low	No	No	No	No	Yes	No	No
**14**	Low	No	No	No	No	Yes	No	No
**15**	High	No	Yes	No	No	Yes	No	Yes
**16**	High	No	Yes	No	No	Yes	No	Yes
**17**	High	No	Yes	No	No	No	No	Yes
**18**	High	No	No	Yes	No	Yes	No	Yes
**19**	Low	No	Yes	No	No	No	No	Yes
**37**	High	Yes	No	Yes	No	No	Yes	Yes
**38**	High	No	No	Yes	No	No	Yes	Yes

**39**	High	Yes	No	Yes	No	Yes	No	No
**40**	High	No	No	Yes	Yes	Yes	No	Yes
**41**	High	No	No	Yes	No	Yes	No	Yes
**42**	High	Yes	No	Yes	Yes	Yes	No	Yes
**43**	High	No	No	No	Yes	Yes	No	Yes
**44**	High	No	No	Yes	No	Yes	No	No
**45**	High	Yes	No	Yes	Yes	Yes	No	Yes
**46**	High	No	No	Yes	Yes	Yes	No	Yes
**47**	Low	No	Yes	No	No	No	No	Yes
**49**	High	No	No	No	No	Yes	Yes	Yes
**50**	High	No	Yes	Yes	Yes	Yes	Yes	Yes
**51**	High	No	No	No	Yes	Yes	No	Yes
**52**	High	No	No	Yes	No	Yes	No	No
**Hydroxanthones derivatives**								
**20**	High	No	No	No	No	No	No	No
**21**	High	No	No	No	No	No	No	No
**22**	Low	No	No	No	No	No	No	No
**23**	Low	No	No	No	No	Yes	No	No
**24**	Low	No	Yes	No	No	No	No	No
**25**	Low	No	Yes	No	No	No	No	No
**26**	Low	No	Yes	No	No	Yes	No	No
**27**	Low	No	No	No	No	Yes	No	No
**28**	Low	No	Yes	No	No	Yes	No	No
**29**	Low	No	Yes	No	No	Yes	No	No
**30**	Low	No	Yes	No	No	Yes	No	No

**31**	Low	No	Yes	No	No	Yes	No	No
**48**	High	No	No	No	No	No	No	No
**53**	Low	No	No	No	No	Yes	No	No
				**Glycosylated and other derivatives**				
**32**	Low	No	Yes	No	No	Yes	No	No
**33**	Low	No	Yes	No	No	No	No	No
**34**	Low	No	Yes	No	No	Yes	No	No
**35**	Low	No	Yes	No	No	Yes	No	No
**36**	Low	No	Yes	No	No	Yes	No	No
